# Identification of *SETBP1* Mutations by Gene Panel Sequencing in Individuals With Intellectual Disability or With “Developmental and Epileptic Encephalopathy”

**DOI:** 10.3389/fneur.2020.593446

**Published:** 2020-12-16

**Authors:** Emanuela Leonardi, Elisa Bettella, Maria Federica Pelizza, Maria Cristina Aspromonte, Roberta Polli, Clementina Boniver, Stefano Sartori, Donatella Milani, Alessandra Murgia

**Affiliations:** ^1^Molecular Genetics of Neurodevelopment, Department of Woman and Child Health, University of Padova, Padua, Italy; ^2^Fondazione Istituto di Ricerca Pediatrica (IRP), Città Della Speranza, Padua, Italy; ^3^Paediatric Neurology and Neurophysiology Unit, Department of Woman and Child Health, University Hospital of Padova, Padua, Italy; ^4^Fondazione Istituto di Ricovero e Cura a Carattere Scientifico (IRCCS), Ca' Granda Ospedale Maggiore Policlinico, Milan, Italy

**Keywords:** SETBP1, ID, epilepsy, epileptic encephalopathy, NGS, gene panel, MRD29

## Abstract

*SETBP1* mutations are associated with the Schinzel-Giedion syndrome (SGS), characterized by profound neurodevelopmental delay, typical facial features, and multiple congenital malformations (OMIM 269150). Refractory epilepsy is a common feature of SGS. Loss of function mutations have been typically associated with a distinct and milder phenotype characterized by intellectual disability and expressive speech impairment. Here we report three variants of *SETBP1*, two novel *de novo* truncating mutations, identified by NGS analysis of an Intellectual Disability gene panel in 600 subjects with non-specific neurodevelopmental disorders, and one missense identified by a developmental epilepsy gene panel tested in 56 pediatric epileptic cases. The three individuals carrying the identified *SETBP1* variants presented mild to severe developmental delay and lacked the cardinal features of classical SGS. One of these subjects, carrying the c.1765C>T (p.Arg589^*^) mutation, had mild Intellectual Disability with speech delay; the second one carrying the c.2199_2203del (p.Glu734Alafs19^*^) mutation had generalized epilepsy, responsive to treatment, and moderate Intellectual Disability; the third patient showed a severe cognitive defects and had a history of drug resistant epilepsy with West syndrome evolved into a Lennox-Gastaut syndrome. This latter subject carries the missense c.2572G>A (p.Glu858Lys) variant, which is absent from the control population, reported as *de novo* in a subject with ASD, and located close to the *SETBP1* hot spot for SGS-associated mutations. Our findings contribute to further characterizing the associated phenotypes and suggest inclusion of *SETBP1* in the list of prioritized genes for the genetic diagnosis of overlapping phenotypes ranging from non-specific neurodevelopmental disorders to “developmental and epileptic encephalopathy” (DEE).

## Introduction

Genotype-phenotype correlations for *SETBP1* gene are extremely complex and clinically relevant. Recurrent missense variants at codons 868-871, forming the critical consensus sequence of the degradation signal, have been associated with the classic Schinzel-Giedion syndrome (SGS, OMIM^*^ 269150) (1), characterized by profound neurodevelopmental delay (NDD), a characteristic facial gestalt, epilepsy, hydronephrosys and multiple congenital anomalies (2). Patients with missense variants near the degradation sequence (codons 862, 867, and 873) exhibit a milder SGS phenotype (3–5) and the clinical overlap with classic SGS phenotype is related to the proximity of the mutated position to the degron (3). Identical *SETBP1* variants were reported as somatic even in several types of myeloid malignancies (6).

Additionally, large scale targeted sequencing within large umbrella cohorts, such as intellectual disability (ID), ASD, and epilepsy allowed to expand the spectrum of *SETBP1*-related disorders (7–11). To date, deletions or truncating mutations resulting in loss of function (LoF) of *SETBP1* protein have been reported in only 16 individuals with a distinct phenotype, the Autosomal Dominant Mental Retardation type 29 (MRD29, OMIM^*^ 616078), characterized by subtle dimorphisms, expressive speech impairment with intact receptive language abilities, decreased fine motor skills, and hyperactivity or autistic traits (7, 8).

Novel clinical criteria based on clinical findings and severity of mutation effects have been proposed in order to classify individual phenotypes caused by SETBP1 alterations including those individuals presenting atypical manifestations (12). However, more clinical and functional knowledge are needed for better genotype-phenotype correlation.

In order to estimate the contribution of *SETBP1* in phenotypes differing from classic SGS, we included *SETBP1* in two NGS-targeted gene panels for the diagnosis of individuals with non-specific neurodevelopmental disorders (NDDs) or developmental and epileptic encephalopathy (DEE). Here, we report a detailed clinical description of two individuals found to carry novel *de novo SETBP1* loss of function variants contributing to reinforce genotype-phenotype correlation for this condition. Additionally, we discuss challenges in the interpretation of variants of uncertain significance (VUSs) in syndromic genes in individuals in whom an overt syndromic diagnosis was not evident. This is the case of the *SETBP1* germline variant p.Glu858Lys, reported as somatic in myeloid malignancies, which we identified in a girl with severe ID and drug resistant epilepsy and found to be *de novo* in her reportedly unaffected mother. Our findings support the inclusion of *SETBP1* in the list of prioritized genes for the genetic diagnosis of overlapping phenotypes ranging from non-specific NDDs to “developmental and epileptic encephalopathy” (DEE).

## Diagnostic Assessment

### Cohort Description

The studied cohort includes 600 individuals with non-specific neurodevelopmental disorder and 56 individuals with developmental epilepsy (DEE), recruited from different Italian public hospitals. As reported by the DSM-5 (Diagnostic and Statistical Manual Disorders, fifth edition), the diagnosis of non-specific NDDs is assigned to an individual “presenting a neurodevelopmental disorder, but not meeting the full diagnostic criteria for one of the known neurodevelopmental disorders.” DEEs are a heterogeneous group of rare epilepsy syndromes that manifest with seizures, behavioral disturbances or EEG abnormalities, that can directly worsen cognition and behavior.

Diverse standardized clinical records specific for NDDs and epileptic cases were used to collect clinical data describing family history and clinical phenotype (auxological parameters, physical features, neurological development, cognitive, and behavioral profile) or presence of associated disorders. Data from electroencephalograms (EEG) and brain magnetic resonance imaging (MRI) were also collected whenever available. We excluded patients reported to have a NDD secondary to premature birth, perinatal hypoxia, peri- or postnatal trauma, neurometabolic disease, or severe infections causing neurological problems. Negative comparative genomic hybridization (CGH) array and Fragile-X test were an inclusion criterion for patients with NDDs.

Written informed consent was obtained from the patient's parents or legal representatives. Family three gave also the consent to photo publication. This study was approved by the Local Ethics Committee, University-Hospital of Padova, Italy.

### Gene Panel Sequencing

DNA was extracted from peripheral blood using Wizard genomic DNA Promega Kit (Promega Corporation). Targeted sequencing was performed using a custom gene panel of 74 NDDs associated genes as previously described in (13). The same approach was used to perform targeted sequencing of a 93 developmental epilepsy gene panel in pediatric epileptic patients; the list of genes is available upon request. Briefly, multiplex PCR-based primer panel was designed with Ion AmpliSeq Designer (Thermo Fisher Scientific); DNA libraries were prepared and enriched using the Ion AmpliSeq Technology (Thermo Fisher Scientific) and sequenced with the semiconductor Ion PGM platform (Thermo Fisher Scientific). The Ion Torrent Software Suite, and related plugins were used to process raw data. Variants were annotated with ANNOVAR (14). Variants were filtered on the basis of their frequency in GnomAD database as well as in our in-house database. We used a high resolution frequency threshold of 0.0002 as recommended by (15, 16). The pathogenicity of filtered variants was evaluated as described in (13). Candidate variants were validated by Sanger sequencing and segregation analysis was performed whenever parents were available. To eliminate non-paternity, we performed an independent microsatellite analysis using 3–10 autosomal microsatellite markers for each family. Microsatellite loci were amplified by PCR using fluorescently labeled primers, and labeled products were analyzed by capillary electrophoresis using the ABI 3100 DNA Analyzer and the Gene Mapper software.

## Results

### Molecular Data

Targeted sequencing of the six *SETBP1* exons (NM_015559.3) in 656 individuals, 600 with NDDs and 56 with epilepsy, allowed to identify a total of 19 variants with allelic frequency <0.0002 in the GnomAD database (one frame shift deletion, one stop gain, nine missense, six synonymous, and two intronic variants). None of the synonymous or intronic variants were predicted to impact splicing by HSF and thus considered as likely benign.

By excluding synonymous variants as well as intronic variants with no splicing predictions, we selected eleven *SETBP1* variants, eight from the 600 NDDs subjects and three from the 56 epileptic patients, for further investigation (Table 1). Two of these variants, the stop codon c.1765C>T (p.Arg589^*^) and the frameshift c.2199_2203del; p.Glu734Alafs19^*^, are novel *SETBP1* variants predicted to result in a truncated protein and were found in two patients of the NDDs cohort (Case 1 and 2). Parental segregation and haplotype analysis determined the *de novo* status of the variants in the two patients.

**Table 1:** Variant curation and classification.

**Gene panel**	**Chr position**	**Mutation effect**	**Mutation**	**Exon**	**Variant segregation**	**Database (dbSNP, COSMIC, Clinvar)**	**gnomAD AC/AN**	**Protein Domain**	**GERP++**	**CADD**	**Intervar**
ID	chr18:42529976:C:T	Missense	c.671C>T; p.Thr224Ile	4	Maternal	//	Absent	//	4.7	19.6	VUS: PM2; BS2
ID	chr18:42530173:G:T	Missense	c.868G>T; p.Ala290Ser	4	Paternal	rs764954143	8/246606	//	4.7	9.6	VUS: PM2; BS2
DEE	chr18:42530507:G:A	Missense	c.1202G>A; p.Arg401Gln	4	NA	rs144407969^*^	22/281166	//	5.8	26.5	VUS: PP3; BS1
ID	chr18:42531070:C:T	Stopgain	c.1765C>T; p.Arg589X	4	*de novo*	//	Absent	AT-Hook1	//	//	Pathogenic: PVS1; PS2; PM2; PP3
ID	chr18:42531498: aAAGAGC:A	Frameshift a deletion	c.2199_2203del; p.Glu734Alafs19^*^	4	*De novo*	//	Absent	SKI	//	//	Pathogenic: PVS1; PS2; PM2; PP3
DEE	chr18:42531877:G:A	Missense	c.2572G>A (p.Glu858Lys)	4	Maternal, *De novo* in the mother	rs1178702025; COSM1666672, COSM1717367; VCV000521296.1	Absent	SKI	6.2	29.9	Likely pathogenic: PS3; PM2; PP3; PP4
ID	chr18:42532328:G:A	Missense	c.3023G>A; p.Arg1008His	4	Maternal	rs760902522	16/282768	//	5.8	24.3	VUS: PP3; BS1
ID	chr18:42532532:C:T	Missense	c.3227C>T; p.Ser1076Leu	4	Paternal	COSM1711241	1/250820	//	5.8	29.1	VUS: PM2; BS2
ID	chr18:42532652:A:G	Missense	c.3347A>G; p.His1116Arg	4	Maternal	rs765137543	15/251190	//	5.9	15.7	VUS: PP3; BS1
DEE	chr18:42533181:C:G	Missense	c.3876C>G; p.Asp1292Glu	4	NA	rs139106261	5/281470	SET-BD	5.3	18.8	VUS: PM2
ID	chr18:42618486:C:A	Missense	c.4037C>A; p.Ala1346Glu	5	NA	rs973611154	Absent	SET-BD	3.1	15.1	VUS: PM2

*Genebank transcript ID (NM_015559.3); total exon count: 6. AC, Allele count; AN, Allele Number: VUS, variant of uncertain significance. NCBI Protein ID: NP_056374.2 (longest isoform) 1596 aa; Protein domains: AT hook 1 (aa 584-596); SKI homologous region (aa 706-917); AT hook 2 (aa 1016-1028); SET binding domain (SET-BD) (aa 1292-1488); AT hook 3 (aa 1451-1463); Repeat domain (aa 1520-1543); Three putative bipartite NLSs are located at amino acids 462–477, 1370–1384, and 1383–1399. GERP++ scoring scheme for residue conservation (score>3.0 indicates conserved positions) (17); CADD (Combined Annotation-Dependent Depletion) pathogenicity prediction tool (18). Variant interpretation based on InterVar criteria was manually adjusted based on our findings (19)*.

* *c.1202G>A; p.Arg401Gln previously identified in a ASD family, paternally inherited in the proband (20). Variants reported in the table have been submitted to LOVD database (https://databases.lovd.nl/shared/genes/SETBP1) corresponding to accession numbers: 315900, 315901, 315902, 316191, 316194, 316195, 316196, 316197, 316198, 316200, 316202*.

Among the nine selected missense variants, five occur at very conserved residue positions and are predicted to have deleterious effects by several computational tools (Table 1). Three of them have a frequency in the general population higher than expected for *SETBP1-*related conditions and thus were considered variants of uncertain significance (VUSs). However, the c.1202G>A; p.Arg401Gln variant, with AF of 0.00007 in Gnomad, has been previously reported in an ASD family (20). Two of the conserved variants, c.2572G>A (p.Glu858Lys) and c.3227C>T (p.Ser1076Leu), were absent or found only once in GnomAD. The two latter variants were also reported as somatic variants (COSM1666672 or COSM1717367, and COSM1711241) in cutaneous malignant melanoma, hematopoietic neoplasms, and in carcinomas (clear cell renal cell, the p.Glu858Lys, and intestinal adenocarcinoma, the p.Ser1076Leu) (Table 1).

The c.3227C>T (p.Ser1076Leu) variant was found in a boy with global developmental delay with a mild cognitive defect, fine motor and expressive language impairment. A previous CGH array investigation identified a *de novo* deletion at 4q21.21 of 334.9 Kb including part of the C4orf22 gene, encoding for the “Cilia and Flagella associated protein” (CFAP299). This gene, recently found to be involved in spermatogenesis in the mouse, is tolerant to loss of function variations (pLI = 0), thus the *de novo* chromosomal alteration was considered of uncertain significance or as a possible further genetic factor contributing to the phenotype. The Ser1076 maps in a conserved region downstream of the AT hook 2 domain and is predicted by Eukaryotic Linear motif (ELM) server as a NEK2 phosphorylation site (consensus binding region 1073-LYLSHT-1078, conservation score 0.68). NEK2 is a cell cycle-regulated kinase that coordinates cell division at multiple levels and is associated with disease progression in non-Hodgkin lymphomas (21). Segregation analysis allowed to establish the presence of the c.3227C>T (p.Ser1076Leu) germinal variant in the paternal DNA sample (**Figure 2**). However, other family members were not available for further segregation. In absence of functional evidence of its pathogenicity, this variant was considered of uncertain significance but we do not exclude it might contribute to the patient phenotype together with other genetic factors.

The c.2572G>A (p.Glu858Lys) variant was identified in a girl (case 3) referred to our center for developmental and epileptic encephalopathy. This variant maps in the SKI homologous region, close to the *SETBP1* hot spot for SGS-associated mutations. Transcriptomic analysis of aCML cells with somatic *SETBP1* variants (including the p.Glu858Lys variant) revealed changes in the expression of genes transcriptionally controlled by TGF-β1 (3, 6). Additionally, the germline c.2572G>A (p.Glu858Lys) variant was previously reported as *de novo* in a subject with ASD (9) and in Clinvar database (VCV000521296.1), in a female with hereditary neurodevelopmental disorder, intellectual disability, hypotonia, and “early onset neurologic disorder.”

Familial segregation and haplotype analysis determined that, in our case, the c.2572G>A (p.Glu858Lys) variant was inherited from the reportedly healthy mother. Due to multiple evidences supporting the pathogenicity of this variant, we extended the analysis to other relatives, allowing to establish the absence of the variant in the healthy proband's sister and its *de novo* status in her mother (**Figure 2**). Sanger sequencing and NGS excluded the presence of mosaicism for this variant in the mother's DNA sample extracted from peripheral blood leukocytes (**Figure 2**). However, other tissues from this individual were not available to further investigate the presence of mosaicism.

### Clinical Assessment

The sequenced NDDs cohort included individuals that during the developmental period presented impairment in the domain of motor skills (79%), cognition (74%), communication (67%), and/or behavior (66%). In 60% of the sequenced individuals, intellectual quotient (IQ) and adaptive skills were evaluated by means of standardized neuropsychological tests and the NDDs patients were classified in mild (IQ 79–89; 45%), moderate (IQ 50–69; 30%), and severe (QI <50; 25%) categories.

For the three individuals carrying pathogenic/likely pathogenic *SETBP1* mutations, clinical features are summarized in Supplementary Table 1, and compared to the phenotypic features of other *SETBP1* patients reported in literature. A detailed description of each of these latter subjects is presented in the following paragraphs going from milder to more severe phenotype.

#### Case 1

Patient 1 is a 13 year-old male born from non-consanguineous parents with no family history of developmental delay. He was born at term via euthocic delivery after an uncomplicated pregnancy. His birth weight was 3.110 kg (25–50th centile) and his length was 52 cm (75–90th centile), OFC was 35 cm (75th centile), and Apgar score 8/10. His physical growth was constant but at the inferior limits (10th centile). He showed psychomotor delay. He said his first word and achieved autonomous walking at 18 months of age. Up to 4 years he could only say 4–5 words but subsequently slowly improved his language abilities. He is attending secondary school with support and has good interactions with his pairs.

At the last examination, at 11 years of age, his cognitive profile showed mild intellectual disability, he still presented language impairment and manifested motor hindrance. He never presented seizures; the EEG was normal while the brain MRI highlighted a thin corpus callosum and a rotated hippocampal tail. At physical examination, dysmetrya of the lower limbs (1 cm), short lingual frenulum, and phimosis, were observed. Subtle facial dysmorphisms were present, which included long face, high forehead, thin upper lip, smooth philtrum, and mild micrognathia. Five café-au-lait spots, fetal pads, and dorsal hirsutism were noted. No other problems were reported, other than color blindness and farsightedness.

His initial diagnostic examinations included conventional karyotyping and CGH array 40 kb resolution), that resulted negative. Targeted NGS analysis of the ID gene panel (13) highlighted the presence of the stop gain c.1765C>T (p.Arg589^*^) variant in *SETBP1* (Table 1). A family segregation study revealed the *de novo* nature of the variant (**Figure 2**).

#### Case 2

Patient 2 is a 17-year-old boy, known to us since the age of 13. The family history is negative for epilepsy and other neurological conditions. Born to unrelated parents at 37 + 5 gestational weeks, after an uneventful pregnancy, by cesarean section due to interruption of contractions after a 19-h labor and fetal tachycardia. Birth weight 2,930 g, Apgar Score IA 9–10. Regular perinatal period. Regular motor development, language delay with first words at 3 years of age. The child presented joint hyperlaxity, microcythemia (also present in the mother), occasional enuresis was reported. Hashimoto's thyroiditis was diagnosed at the age of 13. He always experienced school difficulties, especially in mathematics.

The neuropsychological profile of the patient is characterized by moderate intellectual disability (WISC III at 7 years: QIT 46 QIV 58 QIP 46), medium-severe attention deficit, expressive language disorder, dyspraxia. The boy also suffers from generalized anxiety disorder and shows oppositional-provocative behavioral traits.

He presented epilepsy with three apparently generalized febrile critical episodes, two at the age of 19 months and one at the age of 23 months, with normal EEG. At the age of three reported onset of generalized motor epileptic seizures in apyrexia treated with valproate with good seizure control. At the age of seven, during therapy interruption, he experienced one episode of generalized motor crisis. Currently, still treated with Valproate, he has had no further critical episodes. All EEGs performed since the onset of epilepsy are normal, except for the presence, in one occasion, of unusual figures (slow peaks followed by slow waves of small amplitude) at the level of the parietal regions and the posterior vertex when falling asleep.

Some facial dysmorphisms were observed: long face, with prominent forehead, well-defined and arched eyebrows, upturned nasal tip, short philtrum, tented upper lip, and fleshy lower lip. Several genetic tests were performed: conventional karyotype, CGH array, molecular analysis of the *FMR1* and *SCN1A* genes, all resulted negative. A brain MRI performed at 3 years of age was normal. In 2016, a customized panel targeting 31 early onset epileptic encephalopathy genes (EOEE) was performed and resulted negative.

Targeted NGS analysis of the intellectual disability multigene panel (13) highlighted the presence of the frameshift deletion c.2199_2203del; p.Glu734Alafs19^*^ in *SETBP1* (Table 1). A family segregation study revealed the *de novo* nature of the variant (**Figure 2**).

#### Case 3

Patient 3 is a 12-year-old girl, who first came to our attention at the age of 10. First child of non-consanguineous parents; the mother reported a previous first trimester miscarriage and a family history of unspecified epilepsy. The baby was born at term, with induced delivery, after a pregnancy that reported scarce and hypovalid, fetal movements. Birth weight 3,750 g (75–90th centile), Length 51 cm (75th centile), CC 35 cm (75th centile), Apgar score 8–9. Perinatal period characterized by hypertonia with clenched fists, poor spontaneous movements, frequent and inconsolable crying, hypo-valid sucking, unilateral clubfoot. Severe psychomotor delay was evident since the age of 4 months; the child reached autonomous walking at 3 years of age and was able to pronounce a few words not before 4 years of age. Her language did not subsequently progress. Since 4 months of age, some hand stereotypies were noted.

**Figure 1:**
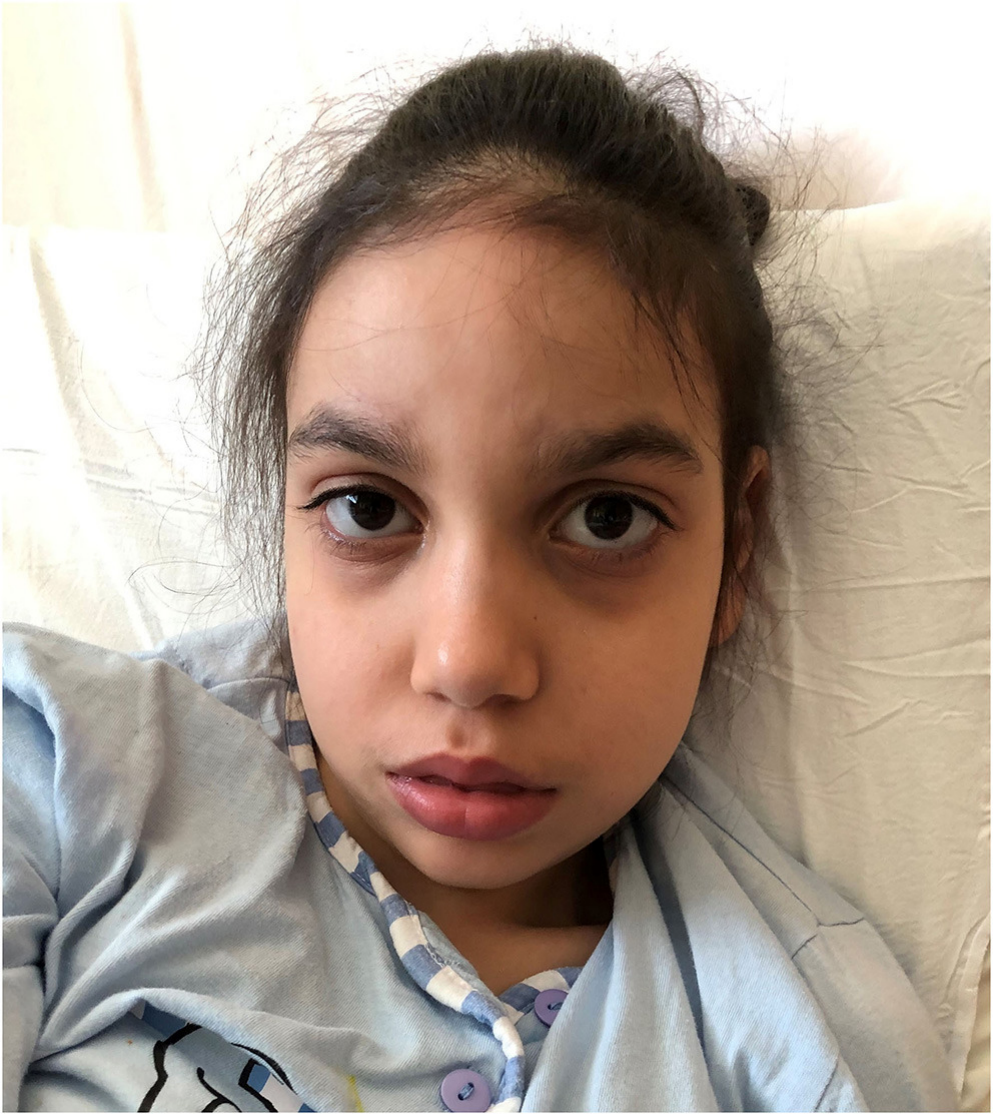
Dysmorphic features of Case 3 carrying the c.2572G>A (p.Glu858Lys) mutation in *SETBP1*. She presents subtle but typical traits of Schinzel-Giedion patients: elongated face with a mild midface retraction, prominent forehead, arched eyebrows, hypertelorism, prominent eyes, shallow orbit, and short neck.

At 10 years of age she showed severe neurological impairment with severe intellectual disability, absent speech, mild ataxia and diffuse muscle hypotrophy, flat feet. She had no sphincter control and no personal autonomy. The neurological examination evidenced progressive microcephaly (CC: 75th centile at birth, 3th centile at 10 years). Dysmorphic features were represented by elongated face with a mild midface retraction, prominent forehead, slightly downslanting palpebral fissures, defined and arched eyebrows, hypertelorism, wide nasal bridge, upturned nasal tip, short philtrum, tented upper lip with fleshier lower lip, short neck (Figure 1). Present dorsal hirsutism.

At the moment she is attending secondary school with full support and seems to show some language improvement. She sometimes seems to show interest in the environment and can slowly execute simple orders. Some stereotypic movements still persist, including eye squinting. Her behavior is overall improved, even if low frustration tolerance persists.

A clinical history of epilepsy was present with onset of West Syndrome at 9 months of life with infantile spasms, treated with ACTH. She was subsequently crisis-free up to 5 years of age. She then started to manifest generalized non-motor crises, such as atypical absence, with subsequent appearance of generalized tonic seizures both in waking and in sleep, and with not better characterized episodes of flexion of the trunk, neck and upper limbs, that often presented in clusters, upon awakening or at night. At the age of 10, she came to our attention with a picture of Lennox-Gastaut encephalopathy treated with pharmacological polytherapy.

The electroencephalographic pattern was characterized, with onset at 9 months, by hypsarrhythmia followed by normal EEG recordings up to 3 years of life. Subsequently multifocal anomalies started to appear, first bilateral occipital and subsequently central-temporal, prevalent on the right hemisphere. From age seven, the EEG showed poorly organized electrical activity with continuous epileptiform anomalies tending to spread, prevalently at the level of the anterior regions.

The child has been treated in the course of the years with pharmacological polytherapy (valproate, clobazam, nitrazepam, levetiracetam, rufinamide, topiramate, vigabatrin, oxcarbazepine, phenobarbital) with poor electroclinical control. Partial clinical benefit has been achieved with valproate, steroid therapy, lamotrigine.

The neuro-radiological picture was characterized by absence of the brain parenchyma alterations, slightly reduced thickness of the corpus callosum and enlarged peri-encephalic liquor spaces, with a small right temporo-polar arachnoid cyst and slight enlargement of the cerebellar sulcus.

The following genetic tests performed in the course of the years resulted negative: sequencing of the *MECP2, ARX, CDKL5* genes, targeted NGS for brain malformations genes (gene list not available), CGH array, MS-MLPA of the Prader Willi/Angelman critical region.

**Figure 2:**
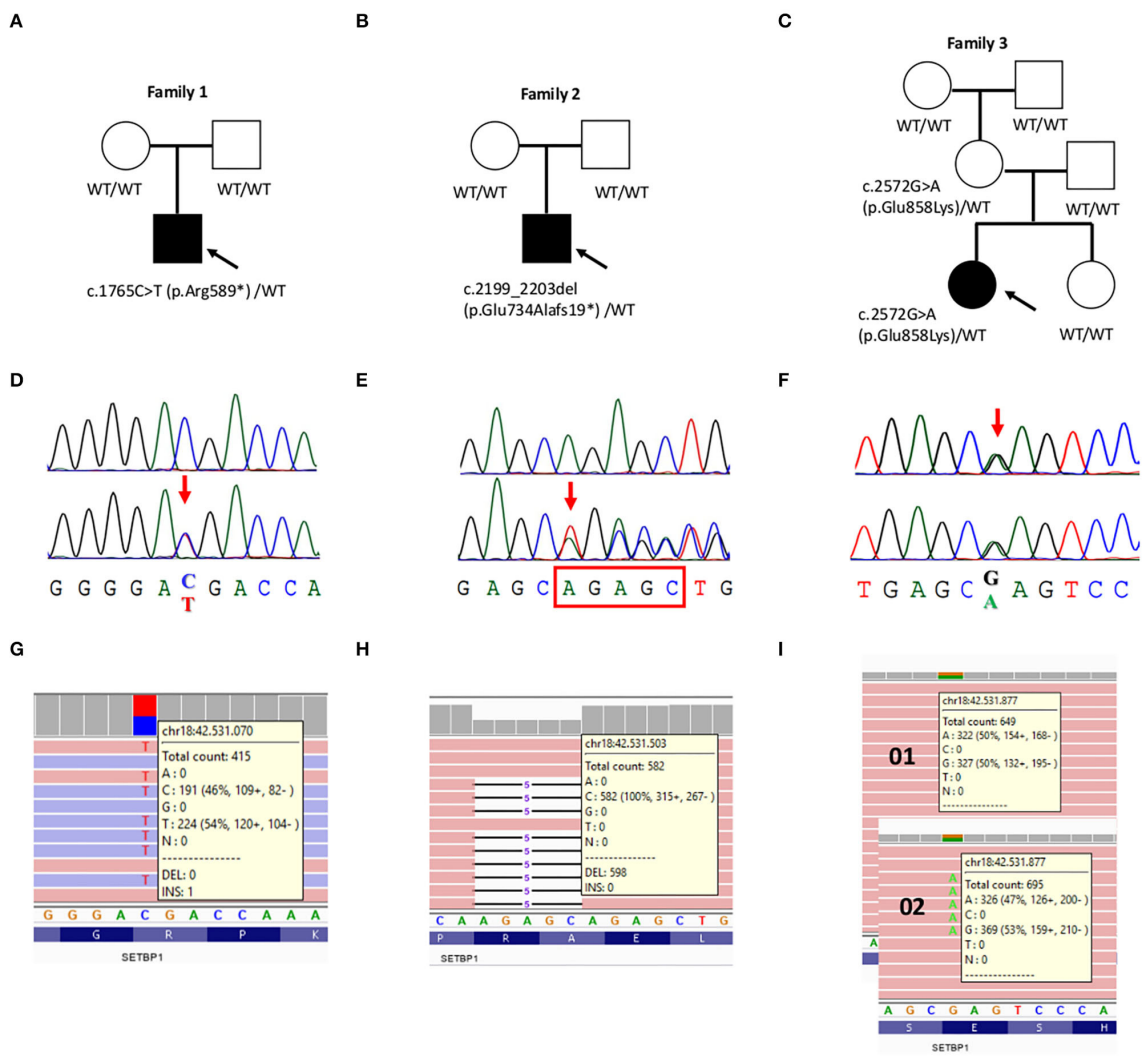
Validation and segregation analysis of the three likely pathogenic *SETBP1* mutations. **(A–C)** Pedigrees of family 1, 2, and 3, respectively. Probands are indicated with an arrow. **(D–F)** Chromatograms of the mutated positions (bottom) compared to the wild-type sequence (upper) **(D,E)** or to the transmitting mother (upper) **(F)**. **(G–I)** Screen shot from the Integrative Genomic Viewer (IGV) visualization showing part of the reads at the mutated positions. **(I)** Possible mosaicism was evaluated in the mother's DNA sample extracted from peripheral blood leukocytes.

A customized panel targeting 93 developmental epilepsy associated genes allowed to identify the maternally inherited *SETBP1* variant, c.2572G>A (p.Glu858Lys) (Table 1 and Figure 2).

## Discussion

Next generation sequencing studies highlighted that *SETBP1* variants known to cause the well-recognized Schinzel-Giedion syndrome (SGS) may be associated with a wide spectrum of clinical presentations (1, 2, 7, 8). Loss of function mutations (LoF) and whole gene deletions cause a distinct and milder phenotype comprising intellectual disability (ID), expressive speech impairment and autism spectrum disorder (ASD) (7, 8, 11, 22). Missense variants close the SGS hot spot have been identified in individuals with a milder SGS phenotype (3, 5). Furthermore, individuals carrying the same *SETBP1* recurrent variant may present very different phenotypes (4, 23).

We included *SETBP1* in two different gene panels used in the diagnostic setting to molecularly characterize 600 individuals referred for non-specific NDDs and 56 cases referred for pediatric epilepsy. Among the 11 novel or very rare *SETBP1* variants we identified, two truncating variants resulted to be *de novo*, and two likely pathogenic missense, previously reported as somatic *SETBP1* variants in hematological malignancies, which we found to be inherited from reportedly asymptomatic parents.

The two LoF mutations predicted to result in truncated proteins, were absent from parental DNA and control population databases, thus have been classified as pathogenic. The stop gain c.1765C>T (p.Arg589^*^) was identified in a boy with mild ID and language impairment, while the frameshift deletion c.2199_2203del; p.Glu734Alafs19^*^ was found in an individual with moderate ID and generalized epilepsy, responsive to treatment. Overall, the phenotype of the two patients of our cohort is consistent with what described for the 16 reported individuals with *SETBP1* LoF point mutations (7, 8, 12, 22, 24).

Along with mild to moderate ID, the most striking features associated with *SETBP1* haploinsufficiency are speech defects in particular as expressive speech impairment or as severe oral dyspraxia (Supplementary Table 1) (8, 12, 22, 24). Deficit in expressive language have been reported both in individuals carrying chromosomal microdeletions in 18q12.3, exclusively including *SETBP1*, as well as in larger 18q chromosomal deletions, and in patients with *SETBP1* point mutations causing haploinsufficiency (25). The two cases we report with LoF SETBP1 variants show language impairment, with expressive language more severely affected. The two novel cases further support the hypothesis that *SETBP1* haploinsufficiency be the main molecular mechanism implicated in the developmental speech deficits in MRD29.

*SETBP1* haploinsufficiency has been associated with distinct dymorphisms including elongated face, characteristic eyebrows and less frequently, low set ears, and cafè-au-lait spots (7). The two patients we report have subtle dysmorphisms with the typical long face and prominent forehead. Case 1 also presents thin upper lip, epicanthic folds and café-au-lait spots and case 2 presents periorbital fullness and a high nasal bridge, both features observed with relatively high frequency in MRD29 patients (Supplementary Table 1). Some of these features remind those of SGS syndrome, such as the upturned nasal tip in case 2. Other features have been observed both in MRD29 and SGS patients, such as the micrognathia seen in case 1 (Supplementary Table 1). It seems that dysmorphic features could be less distinct than suggested for the different phenotypes associated with opposite effects of *SETBP1* variants. Some reported patients with variants in proximity of the degron lack the characteristic gestalt of SGS (3). Moreover, Liu and colleagues reported a patient with a LoF mutation presenting with MRD29 but with dysmorphic features (midface retraction, small upturned nose, and infraorbital groves) typical of SGS (12). Liu et al. proposed a further category to classify SGS patients, which includes those who present typical facial dysmorphisms but lack hydronephrosis and typical skeletal malformations seen in classical SGS patients. In this category may be included the few cases with *SETBP1* mutations presenting with atypical manifestations of SGS (Supplementary Table 1) (2, 12). However, these revised criteria do not well explain the correlation of phenotypes with the type of mutation.

The reported case of a patient carrying a SGS hotspot variant in *SETBP1*, with attenuated SGS and not fulfilling the Lehman diagnostic criteria, suggests that significant phenotypic variability can be found even in patients with variants in the conserved degron motif (4). This could be explained by other genetic risks or protective factors contributing to the disease, as suggested for other neurodevelopmental disorders (26).

This could in fact be the case of the two rare *SETBP1* missense variants, previously reported as somatic in hematological malignancies. that we found inherited from asymptomatic parents.

The germline c.3227C>T (p.Ser1076Leu) was found in a boy with mild developmental delay and expressive speech impairment who carried a *de novo* deletion of uncertain significance at 4q21.21 including part of the C4orf22 gene. This c.3227C>T (p.Ser1076Leu) substitution is predicted to affect the modification of the Ser1076 residue by the cell cycle-regulated kinase NEK2, which coordinates cell division at multiple levels and is associated with disease progression in non-Hodgkin lymphomas (21). The impact of this amino acid substitution might explain its role in cancer. Nevertheless, functional analysis is needed to establish the pathogenic role of this variant in neurodevelopmental conditions.

The other missense c.2572G>A (p.Glu858Lys) variant, identified in a subject referred to our center for developmental epileptic encephalopathy was found to be *de novo* in her reportedly unaffected mother. The c.2572G>A (p.Glu858Lys) has also been reported as a *de novo* germline variant in a patient with ASD (9), and in Clinvar in a female patient with hereditary neurodevelopmental disorder, ID, hypotonia and a non-better specified early-onset neurological impairment. This variant, absent from control population databases, and predicted as pathogenic by several computational tools, maps in close proximity of the conserved degron sequence (aa 868-870). Even if specific functional assays for this variant have not been performed, Acuna-Hidalgo and colleagues demonstrated that mutations in close proximity of the degron cause a modest increase in SETBP1 level and correlate with milder phenotypes (3). Moreover, the same variant has been frequently observed as somatic in patients with atypical chronic myeloid leukemia (aCML) and other hematologic malignancies (6). Experimental evidence of its potential effects on protein function come from transcriptomic analysis of aCML cells carrying the mosaic p.Glu858Lys variant. Compared to control samples, these cells present changes in gene expression, in particular of a subset of genes transcriptionally controlled by TGF-β1 (6).

The patient carrying the c.2572G>A (p.Glu858Lys) had a severe neurodevelopmental disorder but did not present hydronephrosis or cardiac, genital and skeletal anomalies characteristic of SGS. However, the clinical re-evaluation after molecular diagnosis highlighted the presence of subtle but characteristic SGS dysmorphisms, such as midface retraction, prominent forehead, upturned nose, hypertelorism, and other features seen in SGS, such as microcephaly, short neck, hypoplastic nipples, and talipes equinovarus. Additionally, the prominent clinical manifestation of case 3 was epilepsy, which is reported in 95% of the SGS patients with hot spot mutations, and frequently observed also in patients with *SETBP1* haploinsufficiency (3, 7). Our patient had a history of drug resistant West syndrome evolved into a Lennox-Gastaut syndrome poorly controlled by pharmacological polytherapy. West syndrome and seizures that remain refractory to treatment with adrenocorticotropic hormone (ACTH), various antiepileptic drugs, or ketogenic diet have been observed in 25% of the epileptic SGS patients.

Based on these molecular and clinical findings, we consider the c.2572G>A (p.Glu858Lys) variant as a likely pathogenic mutation contributing to the proband phenotype, that might be classified as atypical SGS.

To date all reported *SETBP1* pathogenic mutations have been found to be *de novo*. The finding of likely pathogenic *SETBP1* variants inherited from asymptomatic parents adds challenges in the interpretation of the role of these variants. Several supporting evidences allowed to classify *SETBP1* gene as a high confidence ASD risk gene (https://gene.sfari.org/). For other well-described high impact ASD risk genes, such as *CHD8*, inherited disrupting mutations have been described in families where carrier parents presented with mild NDDs, such as borderline IQ and broader autism phenotypes. Rather than incomplete penetrance, these finding suggested a variable expressivity consistent with a range of ASD manifestations (26).

Although targeted functional analysis is needed to establish the pathogenic role of the identified missense variants in NDD, we cannot exclude that they could contribute, in conjunction with other risk variants, to the phenotype of the patients. On the other hand, asymptomatic carrier parents or individuals presenting with attenuated SGS phenotype may harbor protective genetic variants that dampen a more severe phenotype. In the case of c.2572G>A (p.Glu858Lys), the possibility of incomplete penetrance or variable expressivity, due to somatic mosaicism, since the *de novo* nature of this variant in the mother, could be also hypothesized. However, testing the hypothesis of a somatic mosaicism in other tissues of this individual was not possible.

Further sequencing studies of *SETBP1* in larger cohorts of individuals with broader neurodevelopmental conditions may help to better characterize the contribution of rare inherited *SETBP1* variants or of those affecting the protein function in specific domains which functional role has not yet been established.

## Conclusions

To summarize, we report the clinical details of three individuals carrying *SETBP1* pathogenic mutations detected by NGS screening in two cohorts of individuals referred to our center for non-specific ID and for developmental and epileptic encephalopathy.

The two cases with LoF mutations confirm the phenotype typically associated with *SETBP1* haploinsufficiency. The missense variant p.Glu858Lys, previously reported as a recurrent somatic mutation in hematological malignancies, was found in a patient with severe neurodevelopmental disorder and epileptic encephalopathy. The finding of this as a *de novo* variant in the reportedly asymptomatic mother, suggests the possibility of incomplete penetrance or extreme phenotype variability for *SETBP1* mutations. Our findings contribute to further characterizing the associated phenotypes and suggest inclusion of *SETBP1* in the list of prioritized genes for the genetic diagnosis of overlapping phenotypes ranging from non-specific ID to “developmental and epileptic encephalopathy” (DEE).

## Data Availability Statement

The datasets generated for this study can be found in LOVD database (https://databases.lovd.nl/shared/individuals/SETBP1). Accession numbers can be found within the article and its additional file.

## Ethics Statement

The studies involving human participants were reviewed and approved by Local Ethics Committee, University-Hospital of Padova, Italy. Written informed consent to participate in this study was provided by the participants' legal guardian/next of kin. Written informed consent was obtained from the minor(s)' legal guardian/next of kin for the publication of any potentially identifiable images or data included in this article.

## Author Contributions

EL and AM: conceptualization. EB, RP, MA, and EL: methodology and validation. EL, MA, RP, and EB: formal analysis. EB and EL: data curation. EL, MP, and EB: writing—original draft preparation. AM, EL, and EB: writing—review & editing. EL and AM: funding acquisition. All authors contributed to the article and approved the submitted version.

## Conflict of Interest

The authors declare that the research was conducted in the absence of any commercial or financial relationships that could be construed as a potential conflict of interest.
