# Supplementary material for: Identification of SETBP1 Mutations by Gene Panel Sequencing in Individuals With Intellectual Disability or With “Developmental and Epileptic Encephalopathy”
Source: Front Neurol. 2020 Dec 16;11:593446. doi: 10.3389/fneur.2020.593446 (PMC7772201; doi:10.3389/fneur.2020.593446)
Supplement: Supplementary file 1 [file Table_1.DOCX]

**Supplementary Table S1:** Clinical findings of the reported patients, compared to those reported in individuals with MRD29, atypical or classic Schinzel-Giedion phenotype. Data are presented in percentage for SGS patients and number of patients (n) presenting the associated feature.

| Clinical features | Case 1 | Case2 | Case 3 | MRD29 (n=14) | Atypical SGS (n=5) | SGS (n=44) |
| --- | --- | --- | --- | --- | --- | --- |
| Resources | This Study | This Study | This Study | (Coe et al. 2014) (n=13), (Liu et al. 2018) (n=1) | (Acuna-Hidalgo et al. 2017)(n=3), (Carvalho et al. 2015) (n=1), (Sullivan et al. 2020) (n=1) | (Acuna-Hidalgo et al. 2017) (n=44) |
| Mutation | c.1765C>T; p.Arg589X | c.2194_2198del;p.Glu734Alafs18* | c.2572G>A (p.Glu858Lys) | LoF mutations (13/13; 1/1) | missense close to the degron (n=4); degron mutation (n=1) | Degron missense mutations (aa868-871) (100%) |
| Craniofacial findings |  |  |  |  |  |  |
| SGS gestalt | - | - | +/- | + (n=1) | + (n=3); +/- (n=1) | + (100%) |
| Head | - | - | Microcephaly (progressive) | Brachycephalic (n=1), dolichocephalic (n=1) | Microcephaly (2/5) | Microcephaly 74%; Large anterior fontanelle/sutures (90%) |
| Face | Long face, high forehead | Long face, prominent forehead | Long face, prominent forehead, mild midface retraction | Long face (10/13); forehead (high, prominent, narrow) (n=6) | Forehead (high, prominent, narrow) (n=5); midface retraction (n=5) | Midface retraction (100%); bitemporal narrowing (89%); forehead (high, prominent) (93%) |
| Eyes | - | Periorbital fullness, defined/arched eyebrows | Slightly downslanting palpebral fissures, arched eyebrows, hypertelorism | Ptosis (n=7); palpebral fissures (small, downslanted, short) (n=5); periorbital fullness (n=5); synophrys (n=5); hypertelorism (n=6); infraorbital groove (n=1) | Hypertelorism (n=2); upslanting palpebral fissures (n=1); prominent eyes/shallow orbits (n=2); infraorbital groove (n=1) | Hypertelorism (86%); upslanting palpebral fissures (81%); prominent eyes/shallow orbits (95%); infraorbital groove (97%) |
| Nose | Smooth philtrum | Upturned nasal tip, short philtrum | Wide nasal bridge, upturned nasal tip, short philtrum | Nasal bridge (long, broad, wide, high n=6, low n=1); small nose (n=2); nasal tip (full) (n=3); short, upturned nose (n=1) | Short, upturned nose, depressed root (3/5) | Short, upturned nose, depressed root (98%) |
| Mouth | Thin upper lip | Tented upper lip, fleshy lower lip | Tented upper lip, fleshy lower lip | Thin upper lip (n=3); high palate (narrow) (n=3); wide (n=1) small (n=1); fleshy lower lip (n=1) | Macroglossia (n=1); macrostomia(n=1) | Macroglossia (47%); macrostomia(52%) |
| Chin | Mild micrognathia | - | - | Pointed/long chin (n=3), micrognatia/retrognatia (n=1) | micrognatia/retrognatia (n=1) | micrognatia/retrognatia 97% |
| Ears | - | - | - | Low set ears (n=5), Abnormal helices and pinnae (ears large, protruding) (n=4) | Low set ears (n=1), Abnormal helices and pinnae (ears large, protruding) (n=1) | Low set ears (87%); Abnormal helices and pinnae (ears large, protruding) (97%) |
| Hair | - | - | Dorsal hirsutism | Hirsutism (n=1) hypertrichosis (n=1) | Hirsutism (n=1) | Hirsutism/Hypertrichosis (70%) |
| Neck | - | - | Short neck | - | - | Short neck (91%) |
| Skin | Café-au-lait spots | - | - | Café-au-lait spots (4/13 but one with NF1 mutation) |  | Facial hemangioma (24%) |
| Neuro-developmental anomalies | + | + | + | + | + | + |
| Developmental delay | Mild | Moderate | Severe | Mild-Severe | Moderate (n=2); severe (n=2) | Severe |
| Motor impairment | Fine motor | - (regular) | + (mild ataxia and muscle hypotrophy) | Mild motor delay (n=10), fine motor impairment (8/13), hypotonia in infancy (n=2) | Mild motor delay (n=1) | + |
| Language impairment | + (expressive) | +(expressive, dyspraxia) | + (absent) | Absent or impaired speech (12/13) Receptive better that expressive; Dyspraxia; Use of signs to communicate (n=6/14) | + | + |
| Behavioral impairment | Friendly, low frustration tolerance | Medium-severe attention deficit, generalized anxiety disorder, oppositional-provocative behavior | Quite, low frustration tolerance | Autistic features (6/14); hyperactivity (5/14); ADHD (5/14); aggressiveness (3/14); sleeping disorder (n=2); no sense of pain (n=2); friendly (1/14); quite, apathic (1/14) | Friendly (n=1); short attention span (n=1); self-injurious (n=1) |  |
| Seizures/Epilepsy | - | + (generalized, febrile; generalized motor, in apyrexia) | + (West syndrome, Lennox-Gastaux) | Seizure or EEG abnormalities (5/13) | Seizures (3/5) | Epilepsy (95%); West syndrome (25%) |
| Spasticity/hypertonia | - | - | perinatal period: hypertonia with clenched fists, poor spontaneous movements | - | + (n=2) | + (n=85%) |
| Feeding difficulties | - | - | + | - | - |  |
| Vision impairment | colour blindness and farsightedness. | NA | NA | + (n=4) | + (n=2), | + (80%) |
| Hearing impairment | - | - | NA | + (n=1) | - | + (89%) |
| Brain MRI/CT anomalies | thin corpus callosum, rotated hippocampal tail | - (normal) | reduced thickness of the corpus callosum, enlarged  peri-encephalic liquor spaces, right temporo-polar arachnoid cyst, enlargement of the cerebellar sulcus | No major anomalies | + (n=1 /5) Cortical atrophy or dysplasia | Ventriculomegaly (62%), Underdeveloped corpus (82%), cortical atrophy or dysplasia (55%), choroid atrophy cysts (42%) |
| Congenital anomalies | - | - | Hypoplastic nipples, Talipe(s) equinovarus | inverted nipples (n=1) | Genital (n=1), cardiac defects (n=1) | Hydronephrosis (95%), genital (91%), cardiac(46%), tracheolaringomalacia (50%), Hypoplastic, displaced or inverted nipples (74%), Talipe(s) equinovarus (65%) |
| Skeletal anomalies | fetal pads | joint hyperlaxity | flat feet , unilateral clubfoot | Abnormalities of the extremities (n=6), hyperkyphosis (n=1), hyperlordosis (n=1) | Broad ribs (n=1) | Sclerotic base skull or mastoid (83%), hypoplastic distal phalanges (84%), broad ribs (87%), hypoplastic pubic bones (21%) |

**References**

Acuna-Hidalgo, Rocio, Pelagia Deriziotis, Marloes Steehouwer, Christian Gilissen, Sarah A. Graham, Sipko van Dam, Julie Hoover-Fong, et al. 2017. ‘Overlapping SETBP1 Gain-of-Function Mutations in Schinzel-Giedion Syndrome and Hematologic Malignancies’. Edited by Gregory S. Barsh. *PLOS Genetics* 13 (3): e1006683. https://doi.org/10.1371/journal.pgen.1006683.

Carvalho, Ellaine, Rachel Honjo, Monize Magalhães, Guilherme Yamamoto, Katia Rocha, Michel Naslavsky, Mayana Zatz, Maria Rita Passos‐Bueno, Chong Kim, and Debora Bertola. 2015. ‘Schinzel–Giedion Syndrome in Two Brazilian Patients: Report of a Novel Mutation in SETBP1 and Literature Review of the Clinical Features’. *American Journal of Medical Genetics Part A* 167 (5): 1039–46. https://doi.org/10.1002/ajmg.a.36789.

Coe, Bradley P, Kali Witherspoon, Jill A Rosenfeld, Bregje W M van Bon, Anneke T Vulto-van Silfhout, Paolo Bosco, Kathryn L Friend, et al. 2014. ‘Refining Analyses of Copy Number Variation Identifies Specific Genes Associated with Developmental Delay’. *Nature Genetics* 46 (10): 1063–71. https://doi.org/10.1038/ng.3092.

Liu, Wei-Liang, Zhi-Xu He, Fang Li, Rong Ai, and Hong-Wei Ma. 2018. ‘Schinzel–Giedion Syndrome: A Novel Case, Review and Revised Diagnostic Criteria’. *Journal of Genetics* 97 (1): 35–46. https://doi.org/10.1007/s12041-017-0877-5.

Sullivan, Jennifer A., Nicholas Stong, Evan H. Baugh, Marie T. McDonald, Akihito Takeuchi, and Vandana Shashi. 2020. ‘A Pathogenic Variant in the SETBP1 Hotspot Results in a Forme-Fruste Schinzel-Giedion Syndrome’. *American Journal of Medical Genetics. Part A*, May. https://doi.org/10.1002/ajmg.a.61630.
